# Environmental and Taxonomic Drivers of Bacterial Extracellular Vesicle Production in Marine Ecosystems

**DOI:** 10.1128/aem.00594-23

**Published:** 2023-05-18

**Authors:** Steven J. Biller, Allison Coe, Aldo A. Arellano, Keven Dooley, Samantha M. Silvestri, Jacqueline S. Gong, Emily A. Yeager, Jamie W. Becker, Sallie W. Chisholm

**Affiliations:** a Department of Civil and Environmental Engineering, Massachusetts Institute of Technology, Cambridge, Massachusetts, USA; b Department of Biological Sciences, Wellesley College, Wellesley, Massachusetts, USA; c Science Department, Alvernia University, Reading, Pennsylvania, USA; d Department of Biology, Massachusetts Institute of Technology, Cambridge, Massachusetts, USA; University of Delaware

**Keywords:** *Prochlorococcus*, *Pelagibacter*, *Alteromonas*, *Thalassospira*, *Alcanivorax*, *Marinobacter*, *Dokdonia*, *Polaribacter*, North Pacific Gyre, extracellular vesicles

## Abstract

Extracellular vesicles are small (approximately 50 to 250 nm in diameter), membrane-bound structures that are released by cells into their surrounding environment. Heterogeneous populations of vesicles are abundant in the global oceans, and they likely play a number of ecological roles in these microbially dominated ecosystems. Here, we examine how vesicle production and size vary among different strains of cultivated marine microbes as well as explore the degree to which this is influenced by key environmental variables. We show that both vesicle production rates and vesicle sizes significantly differ among cultures of marine Proteobacteria, Cyanobacteria, and Bacteroidetes. Further, these properties vary within individual strains as a function of differences in environmental conditions, such as nutrients, temperature, and light irradiance. Thus, both community composition and the local abiotic environment are expected to modulate the production and standing stock of vesicles in the oceans. Examining samples from the oligotrophic North Pacific Gyre, we show depth-dependent changes in the abundance of vesicle-like particles in the upper water column in a manner that is broadly consistent with culture observations: the highest vesicle abundances are found near the surface, where the light irradiances and the temperatures are the greatest, and they then decrease with depth. This work represents the beginnings of a quantitative framework for describing extracellular vesicle dynamics in the oceans, which is essential as we begin to incorporate vesicles into our ecological and biogeochemical understanding of marine ecosystems.

**IMPORTANCE** Bacteria release extracellular vesicles that contain a wide variety of cellular compounds, including lipids, proteins, nucleic acids, and small molecules, into their surrounding environment. These structures are found in diverse microbial habitats, including the oceans, where their distributions vary throughout the water column and likely affect their functional impacts within microbial ecosystems. Using a quantitative analysis of marine microbial cultures, we show that bacterial vesicle production in the oceans is shaped by a combination of biotic and abiotic factors. Different marine taxa release vesicles at rates that vary across an order of magnitude, and vesicle production changes dynamically as a function of environmental conditions. These findings represent a step forward in our understanding of bacterial extracellular vesicle production dynamics and provide a basis for the quantitative exploration of the factors that shape vesicle dynamics in natural ecosystems.

## INTRODUCTION

Cells from all domains of life produce small (approximately 50 to 250 nm in diameter), membrane-bound structures that are known as extracellular vesicles. Much of what we know about vesicle release by bacteria is derived from studies of model microbes that are relevant to advances in molecular biology or biomedical research. However, the theater has recently been expanded to examine the microorganisms that serve as the biogeochemical engines of natural ecosystems. We have demonstrated that the globally abundant marine cyanobacterium *Prochlorococcus* releases vesicles that contain a wide range of biomolecules, including lipids, nucleic acids, proteins, and metabolites (1–3). Extracellular vesicles are abundant in the oceans, where they are produced by a diverse suite of marine bacteria, archaea, and eukaryotes from both temperate and polar regions (1, 2, 4–8). Though the precise functional roles of vesicles in marine ecosystems remain unknown, lab and field studies suggest that these structures contribute to many biological processes, including horizontal gene transfer, host-phage interactions, and nutrient exchange (1, 9–11). Vesicle concentrations vary between coastal and oligotrophic waters and throughout the water column (1), but the processes that shape these distributions are not clear.

The production of extracellular vesicles appears to be essentially ubiquitous among bacteria. In Gram-negative bacteria, vesicles that are released by intact, viable cells are largely thought to arise via the “blebbing” of the outer membrane, where small regions of the lipid bilayer protrude away from the cell and eventually separate in the form of a spherical vesicle (12, 13). These membrane protrusions could originate from numerous sources, such as localized membrane changes, uneven membrane synthesis, membrane turgor pressure, or the influence of small molecules on local regions of the membrane (14–18). Processes that are associated with the rotation of sheathed flagella generate extracellular vesicles, as well (19). In addition to membrane blebbing, vesicles can also arise from the reannealing of membrane fragments that are produced by cell lysis events (20–22). Such lytic processes may form vesicles with a composition that is distinct from that of “blebbed” vesicles (23, 24).

Given the underlying complexity and diversity of vesicle biogenesis and release, understanding how vesicle production might be regulated, and even the degree to which it is directly regulated in the first place, represents a challenge. In some Gram-negative pathogens and human commensals, vesicle formation appears to involve some degree of genetic control, though it is not known whether these impacts arise through direct or indirect effects (25–31). Biotic interactions that are mediated by chemical or other signals also impact vesicle production in some microbes (11, 16, 32). Abiotic environmental factors, including stress induced by changes in temperature, nutrient availability, reactive oxygen species, and UV exposure, are further correlated with increased vesicle production (6, 33–37). These examples show the potential for vesicle production to vary in response to intracellular and extracellular conditions, but the magnitude to which such variables affect the release of vesicles by different microbes remains an open question.

Here, we examine some of the factors that are hypothesized to influence vesicle production within marine microbial ecosystems, focusing on potential roles for community composition and abiotic environmental variables. To this end, we measured vesicle production rates along a spectrum of environmentally relevant conditions for a suite of taxonomically diverse and numerically abundant marine bacteria. We further explored the degree to which vesicle production varies among closely related strains of *Prochlorococcus* as well as how vesicle production rates change across gradients of light and temperature. Finally, we examined the depth distribution of vesicles at a site in the oligotrophic North Pacific Gyre to explore how natural vesicle abundances correlate with environmental parameters.

## RESULTS AND DISCUSSION

### Vesicle production rates and sizes differ among marine microbial taxa.

The oceans contain extracellular vesicles that are released by a diverse range of microbes (1). To determine whether marine bacteria vary in their production of vesicles, we measured the net rates of vesicle release by a set of axenic cultured isolates that were chosen to represent the most abundant bacterial phyla in marine systems: Proteobacteria, Cyanobacteria, and Bacteroidetes. These measurements are based on the changes in total small particle concentrations during exponential growth (see Materials and Methods) (Fig. S1 and S2), which presumably reflect mainly vesicles that are produced by “blebbing”, rather than lytic, mechanisms (18, 22).

**FIG 1:**
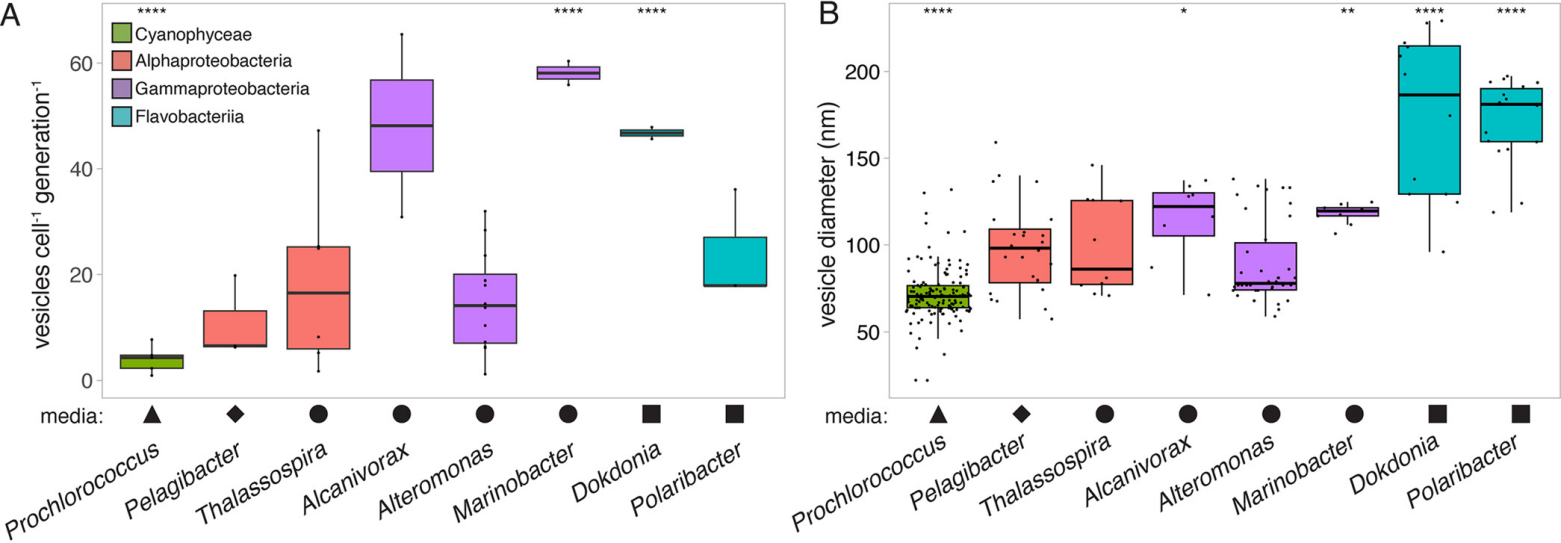
Variation in extracellular vesicle properties across selected marine microbial genera. The plots indicate the measured differences in (A) vesicle production rates and (B) vesicle sizes. The *Prochlorococcus* data represent the median values from five strains that were grown at different light levels (Fig. 2). All cells were grown at 24°C, except *Pelagibacter*, which grew at 22°C. Shapes indicate the seawater-based medium that was required to support strain growth: Pro99 (triangle), ProMS* (diamond), ProMM (circle), or Pro99-PY (square). The colors represent the taxonomic groupings of microbes at the class level: Cyanophyceae (green), Alphaproteobacteria (red), Gammaproteobacteria (purple), or Flavobacteriia (blue). Asterisks indicate strains that differ significantly from the overall mean (Wilcoxon test; *, *P* ≤ 0.05; **, *P* ≤ 0.01; ****, *P* ≤ 0.0001).

The net vesicle release rates in culture, as measured on a per cell per generation basis, varied significantly among the 11 strains that were sampled (one-way analysis of variance [ANOVA], *F*[7,18] = 5.753, *P *< 0.01). The median production rates in cells that were grown at 24°C spanned across an order of magnitude, ranging from approximately 4 vesicles per cell per generation in the cyanobacterium *Prochlorococcus* to approximately 58 vesicles per cell per generation in the heterotroph *Marinobacter*, with other taxa producing vesicles at intermediate values (Fig. 1A). Vesicle production differed significantly by phylum-level groupings (one-way ANOVA, *F*[2,32] = 3.851, *P *< 0.05), though this was driven primarily by a significant difference between Flavobacteria and Cyanobacteria (Tukey’s HSD test, *P *< 0.05). However, significant production rate differences were found among pairs of strains within the same class (*t* test, *P *< 0.05) (Fig. 1A) or genus (discussed below), indicating that vesicle release rates do not vary strictly as a function of cellular taxonomy.

We also found significant differences in the sizes of the vesicles that were released by these microbes (Kruskal-Wallis *H *= 132.62, *P *< 2.2E−16) (Fig. 1B). All strains produced vesicles of heterogeneous size distributions, with *Prochlorococcus* releasing the smallest vesicles (mode of approximately 70 nm in diameter) and members of Bacteroidetes producing the largest (approximately 180 nm) (Fig. 1B; Table 1). Though vesicle size did vary significantly between strains at the phylum level (Wilcoxon test, *P *< 2E−13), we note that the marked overlap in vesicle size distributions precludes us from being able to predict the origin of any individual vesicle, based on this parameter alone.

**TABLE 1:** Summary of extracellular vesicle production rates and diameters, with values indicating the median measurements across all conditions tested in this study

Strain^*a*^	Vesicle production rate median (range) in vesicles cell^−1^ generation^−1^	Vesicle diam median (range) in nm
*Prochlorococcus* MED4 (HLI)	1.2 (0.2–3.5)	65 (53–83)
*Prochlorococcus* MIT9312 (HLII)	2.2 (0.6–8.9)	72 (59–103)
*Prochlorococcus* NATL2A (LLI)	2.5 (0.4–19.0)	67 (58–90)
*Prochlorococcus* MIT9313 (LLIV)	3.9 (0.3–15.4)	74 (58–111)
*Prochlorococcus* MIT1223 (LLVIII)	7.7 (7.7–11.1)	67 (64–73)
*Pelagibacter* HTCC7211	3.5 (0.4–6.6)	98 (58–159)
*Thalassospira* MIT1004	9.3 (2–16.6)	86 (71–146)
*Alcanivorax* MIT1350	48.2 (30.9–65.3)	122 (71–137)
*Alteromonas* MIT1002	16.3 (3.7–28.4)	78 (59–138)
*Marinobacter* MIT1353	35.8 (13.5–58.1)	120 (107–125)
*Dokdonia* MED134	46.8 (45.7–47.9)	186 (96–229)
*Polaribacter* MED152	18.0 (17.9–36.0)	181 (119–197)

a Ecotype or clade/grade designations are provided in parentheses for *Prochlorococcus* strains.

Given that the smallest organisms in our study (*Prochlorococcus* and *Pelagibacter*) also had the lowest vesicle production rates, we wondered whether the total amount of cellular surface area that was available to form membrane vesicles might bound the number of vesicles that can be formed. However, there was no significant relationship between the cellular surface area and the rate of vesicle production (Fig. S3A). The vesicle production rates across strains were, on the other hand, positively correlated with the growth rate (Pearson correlation, *P *< 0.01) (Fig. S4A). On average, each growth rate increase of 1 day^−1^ resulted in approximately 2.5 additional vesicles being released per cell per generation. While correlated, the growth rate only explained approximately 24% of the variation in vesicle release, indicating that other factors must also contribute. The detailed mechanistic connection between the growth rate and the production of vesicles is not yet clear, but we postulate that this could arise in part from changes in the linkage between the outer membrane and the rest of the cell as a consequence of increased rates of cell wall and/or membrane biosynthesis activity. This could, in turn, weaken membrane attachment and create more opportunities for vesicle release.

### Environmental conditions impact vesicle production by marine microbes.

**FIG 2:**
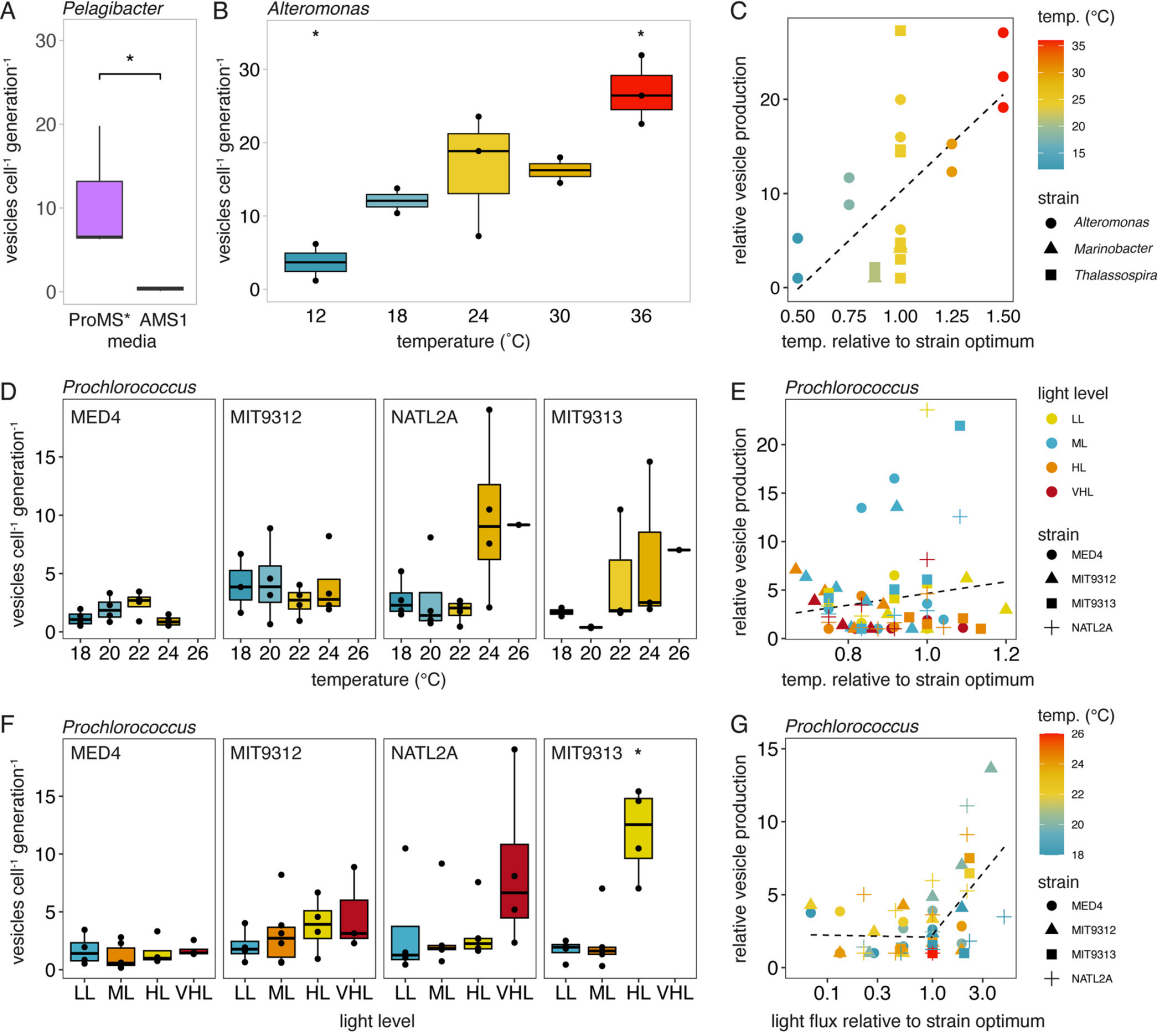
Vesicle production rate variation with changes in environmental factors. (A) *Pelagibacter* vesicle production in two different media. (B) Vesicle release across growth temperatures in *Alteromonas*. (C) Relative changes in vesicle release rates across three different marine heterotrophs, relative to their strain-specific growth optima. (D) Vesicle production rates from four *Prochlorococcus* strains with changes in the growth temperature. (E) Relative vesicle production rates by *Prochlorococcus* strains, compared to the temperature that maximized the growth rate for each strain and light level (optimal = 1). (F) Vesicle production rates from four *Prochlorococcus* strains with changes in light irradiance. LL, low light; ML, medium light; HL, high light; VHL, very high light (Table S1). (G) Relative *Prochlorococcus* vesicle production rates as a function of the light level. Vesicle release is normalized, relative to the minimum measured value for each strain. The light levels are compared to the irradiance that resulted in the maximal growth rate for each strain (optimal = 1). The dashed line in panel C indicates the linear regression fit. The lines in panels E and G represent the change point regression fit. *, *P *< 0.05; Wilcoxon test for panels A and F; *t* test in panel B.

Genetic differences among strains almost certainly play a role in the observed variation in vesicle sizes and release rates across taxa (26), but vesicle production might also vary as a function of environmental factors (33). For instance, though these strains were all grown in seawater-based media under environmentally relevant conditions, some required different media amendments for successful culturing (Fig. 1) (see Materials and Methods). To explore whether the chemical environment might influence vesicle production rates in an abundant oligotrophic marine microbe, we examined cultures of *Pelagibacter* HTCC7211 that had been acclimated to two different media. One culture was grown in a natural seawater-based medium (ProMS*), and the other was grown in a chemically defined artificial seawater medium (AMS1) that differed in the concentrations of trace metals, inorganic nutrients, and organic amendments (see Materials and Methods). *Pelagibacter* grew 3.2× faster in the natural seawater medium than in the artificial seawater medium, and the vesicle release rates were significantly higher, by a factor of approximately 15× in the natural seawater (approximately 6 vesicles per cell per generation in ProMS* versus approximately 0.4 vesicles per cell per generation in AMS1; Wilcoxon test, *P *< 0.05) (Fig. 2A). While we do not know which component(s) of the media are responsible for the difference, this suggests that the local chemical environment can affect vesicle production in oligotrophic organisms. This could occur either by a direct physiological change on the cell or indirectly by influencing the growth rate.

Next, we examined the degree to which changes in temperature influence vesicle release rates. Comparing cultures of *Alteromonas*, which could be grown across a wide range of temperatures, we found that production rates increased with growth temperature (Pearson correlation, *P *< 0.001). The differences were most apparent in the cells that were grown at the lowest and highest temperatures tested (12°C and 36°C), where *Alteromonas* produced vesicles at a significantly different rate from the overall mean (*t* test, *P *< 0.05) (Fig. 2B). Cellular growth rates, while affected by temperature, were not significantly correlated with vesicle production and explained only approximately 7% of the observed variation (Fig. S4B). This suggests that temperature itself was the more important factor. A positive relationship between vesicle production rates and temperature were also noted at physiologically relevant growth temperatures in *Thalassospira* and *Marinobacter* (Fig. S5). Across these three heterotrophs, vesicle production was significantly correlated with temperature (Pearson correlation, *P *< 0.01) (Fig. 2C), with the median rates varying by approximately 8× across the range tested. Though the mechanisms connecting temperature to vesicle release are not specifically known in these organisms, temperature-dependent changes in membrane composition and/or fluidity (18) likely contribute.

### Impacts of light and temperature on *Prochlorococcus* vesicle production.

In *Prochlorococcus* and other abundant marine cyanobacteria, cellular growth and physiology are strongly impacted by both temperature and light levels (38–40). Evolutionary adaptations to these environmental variables also represent deeply divergent traits that structure patterns of diversity within *Prochlorococcus* (41). To expand our examination of how the physical environment impacts vesicle production, we measured the release of vesicles in *Prochlorococcus* cultures that were grown across a landscape of different light irradiances and temperatures. These studies were conducted across five different *Prochlorococcus* strains that were representative of phylogenetically and ecologically distinct high light-adapted and low light-adapted clades (Table 1; Fig. S6). The rates of vesicle production differed significantly among *Prochlorococcus* strains (Kruskal-Wallis *H = *13.88, *P *< 0.01), with the median rates ranging from 1.2 to 7.7 vesicles per cell per generation (Table 1). The production of vesicles by individual *Prochlorococcus* strains was remarkably dynamic, varying by an average factor of approximately 27 between the lowest and highest production rates that were observed in any individual experiment. The median vesicle release rates were lower in high light-adapted *Prochlorococcus* than in low light-adapted strains, and the high light strains also exhibited a smaller range of variation in vesicle release (Table 1). What might account for this? High light-adapted *Prochlorococcus* cells are typically smaller than low light-adapted cells (40), and cell size was positively correlated with the rates of vesicle production within these closely related isolates (Pearson correlation, *P *< 0.01) (Fig. S3B), though it explained only a small amount (approximately 12%) of the variation in *Prochlorococcus* vesicle release.

Looking across all of the *Prochlorococcus* strains and growth conditions tested, light irradiance was significantly associated with the vesicle release rates, while temperature was not (two-way ANOVA, *P *< 0.01). The differences in the growth rates explain only approximately 7% of the variation in vesicle production (Fig. S4C), suggesting that other physiological factors that are influenced by these changes in the abiotic growth environment must drive the phenotype. *Prochlorococcus* responded to the combination of light irradiance and temperature in complex ways, with the differences in the vesicle production rates being most apparent only within a subset of the examined environmental conditions, which further varied among strains (Fig. S6). Vesicle production by the high light-adapted strains (MED4 and MIT9312) did not significantly change as a function of irradiance, and only MED4 showed a significant trend with temperature (two-way ANOVA, *P *< 0.05) across the range examined (Fig. 2D; Fig. S6). In contrast, the low light-adapted strains NATL2A and MIT9313 both exhibited significant variation in production rates with both light and temperature (two-way ANOVA, *P *< 0.05), but this was mostly due to increases in vesicle release at the highest irradiances and temperatures (Fig. 2D and F; Fig. S6). Together, these data suggest that differences in vesicle production patterns may represent another variable that is associated with the ecological differentiation between high light-adapted and low light-adapted *Prochlorococcus*.

### Vesicle release as a response to stress.

Given that the largest changes in vesicle release were associated with increased temperatures or light irradiance, we asked whether this might be correlated with stress responses. We infer that strains growing above the optimal temperature (i.e., at their maximum growth rate) would be experiencing some form of stress. Looking at *Alteromonas*, growth rates decreased at temperatures above 24°C, yet these elevated temperatures had the highest vesicle release rates (Fig. 2B and C). Similarly, the lowest growth temperatures tested, where cold stresses might be experienced, had the lowest vesicle release rates. In contrast, no general relationship between vesicle production and temperature-dependent growth optima was found among *Prochlorococcus* (Fig. 2E; Fig. S7). Whereas previous work in Acinetobacter baylyi found increased vesicle production under temperature stress (42), such a relationship was not found in Pseudomonas aeruginosa (33). Thus, though our data support the hypothesis that vesicle production could be either directly or indirectly affected by temperature stress responses in some microbes, such a connection is not yet apparent and may not be universal.

Next, we looked for associations between vesicle release and light stress in *Prochlorococcus*. Irradiance levels higher than can be processed by photosystems can disrupt the intracellular redox balance, inhibit photosynthesis, or generate reactive oxygen species in cyanobacteria; cells must then activate one or more stress response pathways to repair the damage and/or help to manage the excess energy input (43–45). Across all of the *Prochlorococcus* strains that were examined, vesicle production rates were not influenced by irradiance levels that were below or at those for the optimal (maximized) growth rates (Fig. 2G; Fig. S7). Above this light optimum, when cells are assumed to experience some degree of light stress, production was significantly and positively correlated with relative increases in light (R^2^ = 0.23, linear model, *P *< 0.01) (Fig. 2G). A change point threshold regression analysis corroborated this difference, indicating a significant step increase in vesiculation when light levels were increased above the growth optima at each particular temperature (threshold range: 1 to 2× the optimal light level, *P *< 0.001). No such threshold was found when looking across temperatures (Fig. 2E). This suggests that chronic light stress may directly or indirectly lead to increases in vesicle release in *Prochlorococcus*.

Might vesicle production directly contribute to mitigating light stress, or could it instead reflect a side effect of other physiological changes occurring within the cell? Intracellular oxidative stress, such as that which might arise from high light conditions, increases the number of vesicles produced by Pseudomonas aeruginosa (33), and a similar mechanism could be at play here. *Prochlorococcus* vesicles can contain oxidized metabolites, further raising the possibility that they could aid the cell by removing cellular material that is damaged by oxidative stress (3, 13), including damaged proteins (46). Cyanobacteria can utilize several strategies to dissipate excess light energy during light stress, including modifying their light-harvesting apparatuses, nonphotochemical quenching, and increasing the production of enzymes with antioxidant functions (44). In addition, *Prochlorococcus* can harness excess light energy to power increased levels of carbon fixation and then “dump” the unneeded organic molecules into the extracellular space to maintain the redox balance (43). Although perhaps an indirect metabolic route, the increased synthesis of lipids and the secretion of fixed organic compounds via vesicles might conceivably provide yet another route for the release of the products of overflow metabolism. We can only speculate at this point, but the differences in the light stress responses may explain, in part, the different degree to which light impacts vesicle release by high light-adapted versus low light-adapted *Prochlorococcus*.

### Variation in vesicle size across environmental conditions.

What factors might influence the differences in vesicle sizes that were observed among different marine taxa (Fig. 1B; Table 1)? Cell size was positively correlated with the median vesicle size across the different microbes that were examined here and grown under similar conditions (Pearson’s correlation, *P *< 0.01) (Fig. S8A), explaining 82% of the overall variation. Vesicle sizes were not significantly correlated with the cellular vesicle production rate or the culture growth rate (Fig. S9A and B), indicating that the remaining variation both within and between strains likely reflects physiological differences. Similar results have been seen in host-associated microbes, where vesicle size distributions can differ among closely related strains of the same organism (47).

**FIG 3:**
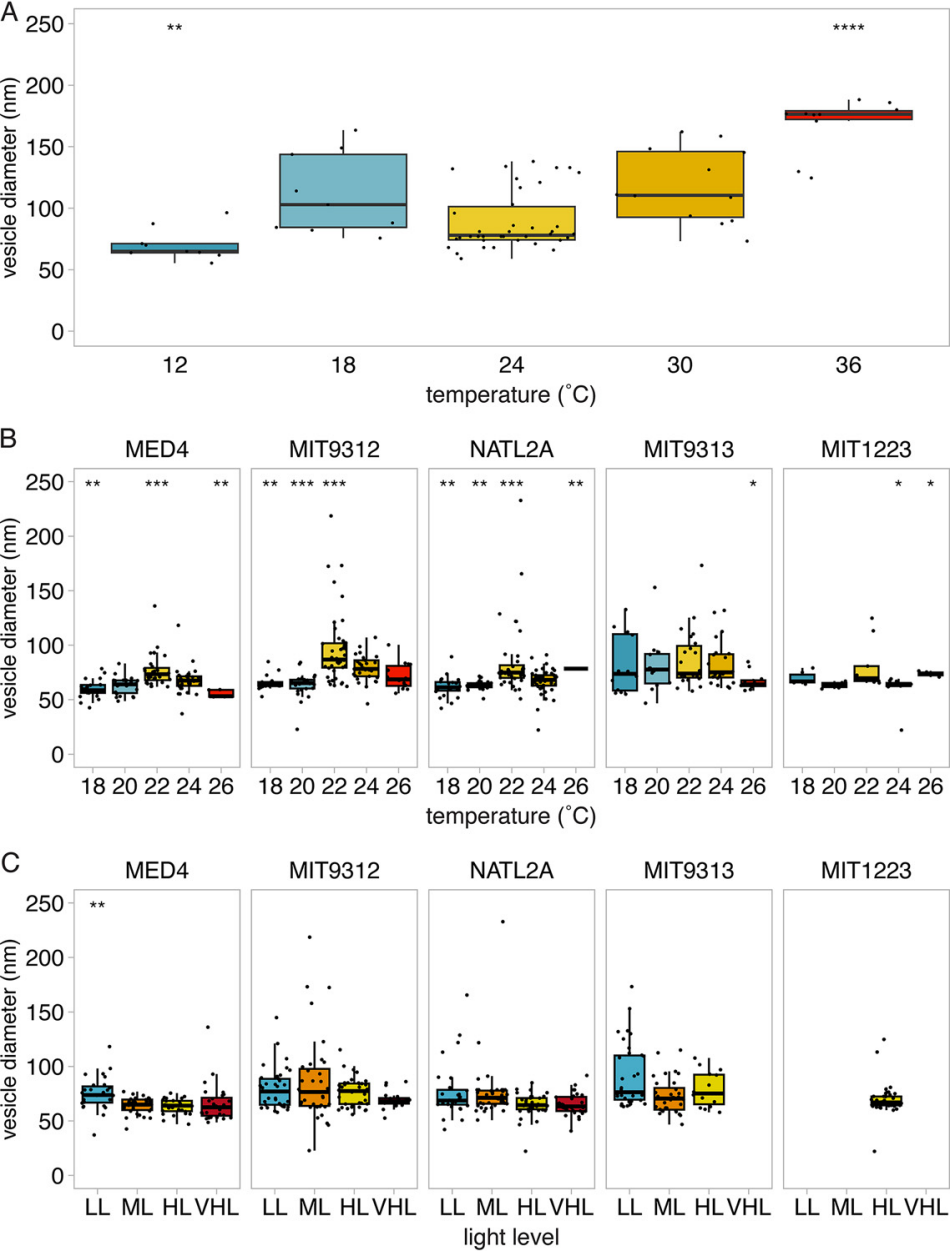
Vesicle size variation with changes in environmental factors. The measured vesicle diameters are shown for (A) Alteromonas macleodii MIT1002 across growth temperatures, (B) *Prochlorococcus* strains across growth temperatures, and (C) *Prochlorococcus* strains grown at different relative light irradiances. LL, low light; ML, medium light; HL, high light; VHL, very high light (Table S1). Asterisks indicate conditions that differ significantly from the overall strain mean diameter (Wilcoxon test; *, *P* ≤ 0.05; **, *P* ≤ 0.01; ***, *P* ≤ 0.001; ****, *P* ≤ 0.0001).

Next, we wondered whether environmental conditions might influence vesicle sizes. Examining *Alteromonas* grown at different temperatures, we found that the vesicle diameter was significantly associated with temperature (Kruskal-Wallis *H *= 38.82, *P* < 7E−8) (Fig. 3A) but not with the cellular growth rate (Fig. S9C). The vesicle size distribution across temperatures followed patterns that were analogous to those that were seen for growth rates. *Alteromonas* vesicles were of similar diameters when grown between 18 and 30°C, but cultures grown at lower temperatures released significantly smaller vesicles, and elevated growth temperatures led to larger vesicles (Wilcoxon test, *P *< 0.01). Vesicle size can be influenced by changes in membrane composition (48); as temperature influences membrane lipid composition (49), temperature-related shifts in membrane composition may influence the resulting curvature and the sizes of the vesicles seen here.

*Prochlorococcus* vesicle size also varied significantly as a function of temperature (Kruskal-Wallis *H *= 107.91, *P *< 2e-16; Fig. 3B) and, to a lesser extent, light intensity (Kruskal-Wallis *H *= 34.89, *P *< 2E−7) (Fig. 3C). As with the changes in vesicle production rates, the differences in the sizes of the vesicles differed among individual *Prochlorococcus* strains; high light-adapted strains, such as MED4 and MIT9312, released the largest vesicles at 22°C, whereas size variation trends were less discernible in low light-adapted strains (Fig. 3B). *Prochlorococcus* vesicle size decreased with increased light flux (Pearson correlation, *P *< 1E−5), even at levels well below the optimum (Fig. 3C). Thus, environmental factors represent another variable, along with factors such as the growth phase (50, 51), that can influence the sizes of the vesicles that are released by a given strain.

### Vesicle production as an investment of cellular resources.

Might taxa differ in the relative investments of resources that they put into vesicles? As an initial approach to addressing this question, we calculated how much membrane material is required to produce the median number of vesicles released per generation, compared to each cell’s total surface area. Despite differences in cell sizes, vesicle diameters, and vesicle production rates, we estimate that most strains, including *Prochlorococcus*, *Pelagibacter*, and *Alteromonas*, release vesicles equivalent to a median of approximately 3 to 6% of their cell surface lipid membranes per generation (Fig. S10). *Alcanivorax* and *Marinobacter*, on the other hand, released vesicles equivalent to approximately 40% of their surface area per generation (Fig. S10). Forming a vesicle of a given size requires proportionately less membrane material from larger cells than from smaller cells, but increased production rates in larger cells can lead to substantial total investments. Given the dynamic range of vesicle production rates, and, thus, the relative cellular investments, cells must increase membrane synthesis under certain growth conditions, regardless of their size. Understanding how these membrane-focused estimates relate to the total amount of cellular material released in heterogenous vesicle populations is an interesting area for future research. For instance, changes in growth conditions will affect the sizes of different cellular pools, growth rate-dependent changes in membrane structure and turnover may influence the amount of this material that can ultimately enter the vesicle lumen, and shifts in vesicle size will bound the maximum capacity of each packet. The costs and benefits of changing vesicle production rates under a given environmental context, especially considering that other cellular resources, such as proteins and nucleic acids, will also be released, are clearly complex (52).

### Abundances of vesicle-like particles across environmental gradients in the North Pacific.

**FIG 4:**
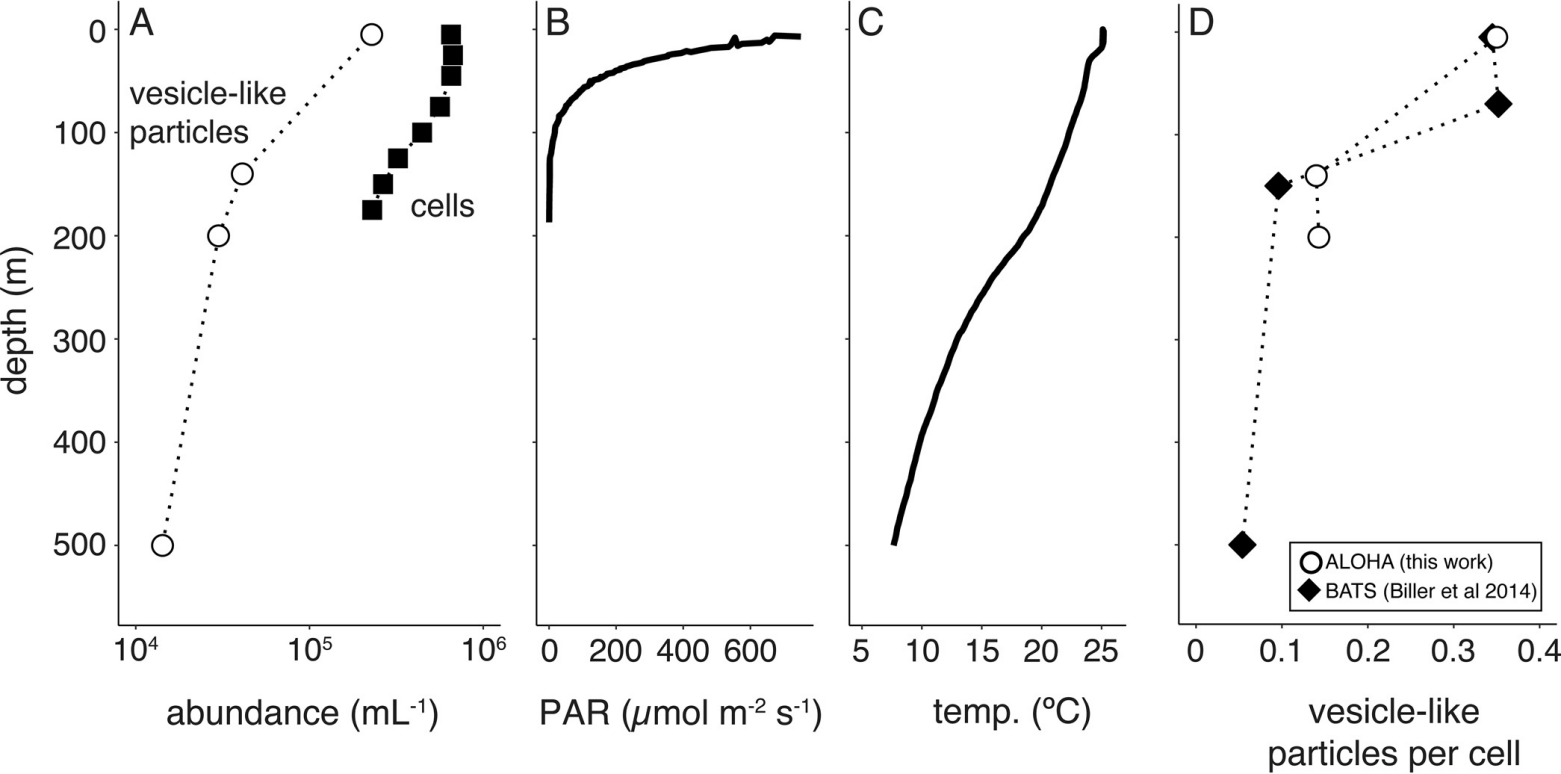
Distribution of vesicle-like particles in the North Pacific Subtropical Gyre. (A) Concentration of vesicle-like particles (open circles) and cells (bacteria and eukaryotes, filled squares) from Station ALOHA in June of 2014. Contextual environmental data are shown for (B) photosynthetically active radiation (PAR) levels and (C) temperature. (D) Relative ratio of vesicle-like particles to cells from Station ALOHA (this work, open circles) and the Bermuda-Atlantic Time Series Station (BATS, collected in December of 2012, filled diamond) (1).

We have observed that extracellular vesicle concentrations decrease with depth at an oligotrophic site in the Atlantic Ocean (1). To explore whether this is generalizable to other regions of the ocean, we measured vesicle abundances at Station ALOHA in the North Pacific subtropical gyre (Fig. 4A). As at the Atlantic site, vesicle-like particles were most abundant near the surface, with their concentration decreasing by approximately an order of magnitude over the upper 500 m. Vesicle-like particle concentrations in this set of Pacific Ocean samples were of the same magnitude as those seen in the Atlantic, and they also declined with depth to a similar degree. This overall congruence of results across different oceans, seasons, and years provides an early indication that these patterns might be representative of those of mid-latitude oligotrophic waters.

The decline of vesicle-like particles with depth broadly mirrors overall trends in light irradiance and temperature *in situ*, both of which are greatest at the surface and also decline with depth (Fig. 4B and C). The changes in vesicle concentrations do not simply reflect cell abundances, as in both the Pacific and Atlantic data, we found proportionally more vesicles per cell at the surface than in deeper samples (Fig. 4D). This is broadly consistent with the culture data indicating a role for environmental factors affecting vesicle abundances. Both the increased irradiance and temperature at the surface would be expected to generally increase the net per-cell vesicle production rates. Differences in community composition likely contribute to these patterns as well. For instance, whereas the low light-adapted *Prochlorococcus* found toward the base of the euphotic zone generally produced more vesicles per cell than did the surface-dwelling, high light-adapted strains, they are much less abundant, overall (53). Thus, they might be predicted to have a lower proportional impact on overall *in situ* vesicle abundances.

### Conclusions and future directions.

Here, we show that extracellular vesicle production rates differ among marine microbes, quantify these differences, and highlight the dynamic nature of vesicle production by individual bacterial cells. The taxonomic origin of extracellular vesicle populations in a given location will be influenced by a complex set of factors that involves differences in cellular community structures as well as the vesicle production rate of each taxon under local environmental conditions. The relationship between the growth rate and vesicle production further suggests that rapidly growing cells within a community might have a relatively pronounced contribution to local vesicle populations, both as a function of increased cell numbers and increased per-cell release. The degree to which genetic adaptations or other factors, such as phage lysis (20, 21), may further influence vesicle release remains an open question.

The net vesicle production data generated here represent a first step toward the development of a quantitative understanding of bacterial vesicle production and vesicle distributions in the marine environment, which are important factors in understanding the ecological functions of these particles. Future efforts should focus on methods by which to deconvolute net vesicle production measurements into separate terms of vesicle release and cellular fusion/degradation rates. Community vesicle production data in field samples, when analyzed in conjunction with *in situ* vesicle concentrations across space and time, may allow us to estimate vesicle loss rates, which are currently completely unconstrained.

Changes in vesicle production as a function of environmental shifts will undoubtedly influence the ecological functions that they carry out, both locally and when viewed across broad gradients throughout the water column. In general, vesicle-cell interactions would be expected to occur more frequently in the upper water column, where the vesicle to cell ratio is greatest, and decrease with depth. The functional outcomes of these interactions in different regions will be complex due to environmental impacts on the sizes and contents of the vesicles. For example, differences in vesicle size are associated with changes in both proteomic (54) and DNA content (55) in some microbes. As *Prochlorococcus* vesicle size changes with temperature, perhaps the proportional rates of horizontal gene transfer or extracellular enzyme activity may also differ with depth. Vesicle size also likely influences diffusion rates, representing yet another factor that may affect interaction dynamics. Overall, these data raise a host of further questions about the potential for shifts in vesicle ecological functions across gradients of light, temperature, and nutrients in the water column.

## MATERIALS AND METHODS

### Culturing conditions.

Axenic *Prochlorococcus* cells were grown in 0.2 μm filtered and autoclaved Sargasso Sea water amended with Pro99 nutrients (56). Cultures were grown on a 14:10 h light:dark cycle at temperatures ranging from 15°C to 26°C and at four light levels: very high light (VHL), high light (HL), medium light (ML), and low light (LL), with the specific values being determined by the light tolerance of each strain (Table S1). Culture axenicity was regularly verified by using a suite of purity test broths (MPTB, ProAC, ProMM) (57–59) and by flow cytometry. Cultures were maintained at the indicated temperature and light intensity for at least 12 generations (approximately 4 complete transfers) and were not sampled until the culture reached balanced growth. Growth was routinely monitored by culture fluorescence with a 10 AU or TD700 Fluorometer (Turner Designs), and the growth rates were calculated via exponential regression from the log-linear portion of the growth curve.

*Alcanivorax* MIT1350 was isolated from a *Prochlorococcus* enrichment culture that was derived from a “small cell, low nucleic acid” population that was flow-sorted from a 150 m water sample that was collected at Station ALOHA (22.75°N, 158°W) in the North Pacific in 2013. This strain was isolated by plating onto ProMM agar plates and by repurifying the colony twice on agar. Following isolation, *Alcanivorax* MIT1350, Alteromonas macleodii strain MIT1002 (60), *Thalassospira* strain MIT1004 (2), and *Marinobacter* MIT1353 (61), were maintained in liquid ProMM medium. The genome sequence of *Alcanivorax* MIT1350 is available at the IMG database (62) under Genome ID 2681813576.

*Pelagibacter* sp. HTCC7211, which is a member of the abundant, warm-water surface-dwelling Ia.3 ecotype of the SAR11 bacterial clade (63), was cultured in either a defined artificial medium (AMS1) (64) or Sargasso seawater-based ProMS* (ProMS medium [61] modified to have vitamin concentrations equal to those of AMS1, plus 10 μM methionine, 50 μM glycine, and 50 μM pyruvate). *Dokdonia* MED134 (65) and *Polaribacter* MED152 (66) were grown in Pro99 media supplemented with 5 g L^−1^ peptone and 1 g L^−1^ yeast extract (BD Difco), herein called “Pro99-PY”. All heterotrophs were routinely maintained at 24°C, except for HTCC7211, which was maintained at 22°C.

### Cell and vesicle enumeration.

All measurements were made on biological triplicate cultures of strains growing under the indicated conditions. At each time point sampled, cells were preserved for flow cytometry via fixation in 0.125% glutaraldehyde, flash frozen in liquid nitrogen, and stored at −80°C until analysis. Vesicles were sampled by collecting 1 mL of culture, filtering it through a 0.22 μm Supor syringe filter (Pall), and storing it at −20°C until analysis. *Prochlorococcus* cells were enumerated using a Guava easyCyte 12HT flow cytometer (Luminex). The heterotrophs were stained with 1× SYBR green I (Molecular Probes) for 1 h and were then counted using either an easyCyte 12HT or a ZE5 flow cytometer (Bio-Rad). *Prochlorococcus* cell sizes were normalized to 2 μm beads (Polysciences). Cells were excited with a blue 488 nm laser and analyzed for red fluorescence (692/40 nm), green fluorescence (530/40 nm), and size (forward scatter). The cell lengths were determined by imaging >500 cells on a Zeiss Axioskop microscope that was equipped with a 100× lens objective and a Nikon D90 digital camera. Cell lengths were determined using ImageJ and were calibrated using a 1 mm/0.01 mm scale stage micrometer (Meiji Techno).

Small particle samples were collected at two or more time points during the exponential growth phase. The particles were enumerated via a nanoparticle tracking analysis using a NanoSight LM10HS instrument (Malvern/NanoSight) that was equipped with a LM14 blue laser module and NTA software V3.1. 3 technical replicate videos (60 s each) were collected from each sample using a camera level setting of 11. Samples containing >80 particles per frame were diluted with clean seawater media to a final concentration of between 20 and 80 particles per frame. The sample chamber was thoroughly flushed with 18.2 MΩ cm^−1^ water (Milli-Q; Millipore) between samples and visually examined to ensure that no particles were carried over between samples. Videos were analyzed using the NTA V3.1 software package with a threshold of 1. The mode size values were taken to represent the vesicle diameter from each population so as to reflect the most abundant size class and minimize the impact of outliers in the tails of the distribution.

Based on prior electron microscopy data from *Prochlorococcus* cultures (2), we operationally define vesicle concentrations as being equivalent to the total number of particles between 50 and 250 nm in diameter in the samples. Media blank controls were routinely run for each batch of media used, and any background concentration was subtracted. Our current methodology cannot rule out that some fraction of particles could be phage or other structures of similar sizes, such as lipoproteins, and not vesicles. To assess the potential contribution of prophage-like structures to the particle counts, we first manually examined the genome sequence of each strain and found no evidence for the presence of gene transfer agents in these organisms (based on BLASTp searches for homology to the Rhodobacter capsulatus gene transfer agent sequences RCAP_rcc01682 to RCAP_rcc01698). Prophage analyses using PHASTER (67) indicated the presence of potential prophage regions in the heterotroph strains MIT1002, MIT1004, MIT1350, MIT1353, and MED134, though all of them were classified as “incomplete” or “questionable”, with none being classified as “intact”. Most heterotroph cultures were grown under *in situ* relevant conditions that were not expected to lead to significant prophage induction, though this cannot be ruled out. While one set of MIT1002 cultures was grown under elevated temperature conditions that might have been considered stressful, all of the samples were collected from exponentially growing cells, and we did not observe any notable shifts in the distributions of particle intensity or size within each time course, nor did we observe other evidence of lytic phage activity in the cultures. A small number of samples containing abnormally high particle counts (defined as containing >10× higher concentrations than other replicates of that time point and/or the surrounding time points) or in which large, highly refractive particles (which mask the underlying particle counts) were present were removed from the final data set. Such samples were scattered across different strains, conditions, and time points, and the sources of these contaminants are unknown. Their occurrence did not follow any obvious systematic patterns. We hypothesize that they could have arisen from manufacturing variations or from defects in the individual filters, plastic tubes used to store the samples, or plastic syringes used in fluid handling.

### Vesicle production and surface area calculations.

We measured the net vesicle production rates (i.e., the number of vesicles formed, minus any vesicles that are degraded, fuse with a cell, or are otherwise lost) as the average number of vesicles produced on a per cell, per generation basis, based on averaged cell and vesicle measurements from all of the biological replicates at each time point. These calculations are based on a model that incorporates the relative rates of change in the cell and vesicle concentrations in pure cultures over time, as detailed in (1). To better account for the variation in slope across the time course, we computed the average number of vesicles that was produced per cell, per generation between each successive pair of time points that was obtained during the exponential growth phase.

The surface area of vesicles from an individual strain was calculated assuming that vesicles are spherical structures with a diameter equivalent to the measured mode value. The cellular surface areas were based on microscopy measurements and literature values (68, 69). *Prochlorococcus* was estimated to be spherical, and the surface areas of other cells were estimated as cylinders with a hemisphere on each end. The relative cellular investment in vesicles per generation was defined as (individual vesicle surface area · vesicle production rate per cell per generation/cell surface area).

### Statistics.

All of the statistical analyses were performed in R (V 4.0.2), and the plots were produced using the “ggplot2” package (70). The data sets were tested for normality using the Shapiro-Wilk test, and nonparametric methods were used where appropriate. Significance values for all multiple pairwise comparisons were corrected using the Benjamini-Hochberg procedure. An analysis of variance [ANOVA] of the vesicle production rate data was performed on the log-transformed values. Model selection for the two-way ANOVAs was carried out using the Akaike information criterion metric, as implemented in the “AICcmodavg” R package (71). The change point threshold regression analysis was carried out using the “chngpt” R package (72). All of the linear regression analyses were carried out using Model I regressions, and two-tailed *t* tests were used.

### Field sampling.

The seawater samples for the vesicle analysis were collected from Station ALOHA on cruise HOT263 (June of 2014). At each depth, we collected 100 to 175 L by using a Niskin bottle array, and we concentrated the <0.2 μm fraction via tangential flow filtration, following previously described protocols (1). The vesicles were enriched across an Optiprep density gradient (Iodixanol; Sigma-Aldrich), as previously described (1). The field particle concentrations were extrapolated from Nanosight measurements of the concentrated, gradient-purified fractions that contained the highest abundance of vesicles (between approximately 1.14 to 1.19 g/mL). We note that particle losses are incurred during the sample processing and analysis pipeline. Thus, these *in situ* measurements should be considered to be lower-bound estimates. The cellular abundance, PAR, and temperature values were obtained from the Hawaii Ocean Time-series program (https://hahana.soest.hawaii.edu/hot/hot-dogs/interface.html). As vesicle-like particles could be produced by microbes from all domains of life, the cell abundances represent the combined counts for cyanobacteria, heterotrophic bacteria, and eukaryotes from the indicated depths.
