# Supplementary material for: Environmental and Taxonomic Drivers of Bacterial Extracellular Vesicle Production in Marine Ecosystems
Source: Appl Environ Microbiol. 2023 May 18;89(6):e00594-23. doi: 10.1128/aem.00594-23 (PMC10304870; doi:10.1128/aem.00594-23)
Supplement: Supplemental file 1 — Supplemental material. Download aem.00594-23-s0001.pdf, PDF file, 2.3 MB [file aem.00594-23-s0001.pdf]

**TABLE S1** Steady state growth rates during exponential phase in axenic *Prochlorococcus* cultures (± SD) at each indicated temperature and light level.

| | | | | Temperature (˚C) | | | | | | | | | | |
|---|---|---|---|---|---|---|---|---|---|---|---|---|---|---|
| strain | ecotype/ clade | light treatment | light level (µmol photons $m^{-2}$ $sec^{-1}$) | 15 | 16 | 18 | 20 | 22 | 24 | 26 | 27 | 28 | 29 | 30 |
| MED4ax | high-light adapted I | VHL | 144±4 | 0.00 ± 0.00 | 0.36 ± 0.02 | 0.37 ± 0.03 | 0.45 ± 0.01 | 0.48 ± 0.00 | 0.47 ± 0.01 | 0.00 ± 0.00 | - | - | - | - |
| MED4ax | high-light adapted I | HL | 76±1 | 0.00 ± 0.00 | 0.31 ± 0.01 | 0.36 ± 0.01 | 0.45 ± 0.01 | 0.48 ± 0.01 | 0.64 ± 0.01 | - | 0.34 ± 0.03 | 0.00 ± 0.00 | - | - |
| MED4ax | high-light adapted I | ML | 40±1 | 0.00 ± 0.00 | 0.25 ± 0.01 | 0.33 ± 0.00 | 0.38 ± 0.01 | 0.38 ± 0.00 | 0.50 ± 0.01 | 0.45 ± 0.01 | 0.19 ± 0.00 | 0.00 ± 0.00 | - | - |
| MED4ax | high-light adapted I | LL | 11±1[a] | 0.00 ± 0.00 | 0.14 ± 0.00 | 0.20 ± 0.01 | 0.23 ± 0.00 | 0.24 ± 0.00 | 0.28 ± 0.01 | 0.00 ± 0.00 | - | - | - | - |
| MIT9312ax | high-light adapted II | VHL | 144±4 | - | - | 0.00 ± 0.00 | 0.31 ± 0.01 | 0.49 ± 0.00 | - | - | 0.52 ± 0.02 | 0.64 ± 0.00 | 0.00 ± 0.00 | - |
| MIT9312ax | high-light adapted II | HL | 76±1 | - | 0.00 ± 0.00 | 0.30 ± 0.01 | 0.40 ± 0.01 | 0.41 ± 0.00 | 0.62 ± 0.01 | 0.71 ± 0.01 | 0.79 ± 0.00 | 0.68 ± 0.01 | 0.45 ± 0.01 | 0.00 ± 0.00 |
| MIT9312ax | high-light adapted II | ML | 40±1 | - | 0.00 ± 0.00 | 0.36 ± 0.00 | 0.40 ± 0.00 | 0.46 ± 0.01 | 0.54 ± 0.01 | 0.70 ± 0.01 | 0.65 ± 0.01 | 0.55 ± 0.00 | 0.41 ± 0.00 | 0.00 ± 0.00 |
| MIT9312ax | high-light adapted II | LL | 11±1 | - | 0.00 ± 0.00 | 0.24 ± 0.00 | 0.32 ± 0.00 | 0.29 ± 0.00 | 0.31 ± 0.00 | 0.25 ± 0.02 | 0.25 ± 0.00 | 0.21 ± 0.00 | 0.00 ± 0.00 | - |
| NATL2Aax | low-light adapted I | VHL | 96±1 | 0.00 ± 0.00 | 0.09 ± 0.00 | 0.28 ± 0.05 | 0.36 ± 0.01 | 0.44 ± 0.00 | 0.53 ± 0.01 | 0.00 ± 0.00 | - | - | - | - |
| NATL2Aax | low-light adapted I | HL | 45±1 | 0.00 ± 0.00 | 0.15 ± 0.00 | 0.26 ± 0.00 | 0.40 ± 0.01 | 0.48 ± 0.00 | 0.58 ± 0.01 | 0.00 ± 0.00 | - | - | - | - |
| NATL2Aax | low-light adapted I | ML | 20±1 | 0.00 ± 0.00 | 0.21 ± 0.00 | 0.30 ± 0.00 | 0.38 ± 0.00 | 0.47 ± 0.00 | 0.54 ± 0.01 | 0.46 ± 0.00 | 0.00 ± 0.00 | - | - | - |
| NATL2Aax | low-light adapted I | LL | 10±0.5 | 0.00 ± 0.00 | 0.19 ± 0.00 | 0.26 ± 0.01 | 0.30 ± 0.00 | 0.34 ± 0.00 | 0.37 ± 0.00 | 0.00 ± 0.00 | - | - | - | - |
| MIT9313ax | low-light adapted IV | HL | 45±1 | - | - | - | 0.00 ± 0.00 | 0.38 ± 0.00 | 0.37 ± 0.02 | - | 0.24 ± 0.02 | 0.00 ± 0.00 | - | - |
| MIT9313ax | low-light adapted IV | ML | 20±1 | - | 0.00 ± 0.00 | 0.26 ± 0.00 | 0.40 ± 0.01 | 0.41 ± 0.00 | 0.47 ± 0.00 | 0.42 ± 0.01 | 0.42 ± 0.00 | 0.00 ± 0.00 | - | - |
| MIT9313ax | low-light adapted IV | LL | 10±0.5 | 0.00 ± 0.00 | 0.16 ± 0.00 | 0.26 ± 0.00 | 0.34 ± 0.00 | 0.32 ± 0.00 | 0.36 ± 0.00 | 0.35 ± 0.00 | 0.31 ± 0.01 | 0.00 ± 0.00 | - | - |
| MIT1223ax | low-light adapted VIII group | HL | 20±1 | - | 0.00 ± 0.00 | 0.23 ± 0.01 | 0.29 ± 0.01 | 0.31 ± 0.00 | 0.38 ± 0.01 | 0.36 ± 0.01 | 0.00 ± 0.00 | - | - | - |

[a]This strain was unable to grow at the indicated light level at 24 ˚C, and was instead grown at 17±0.5 µmol photons $m^{-2}$ $s^{-1}$.

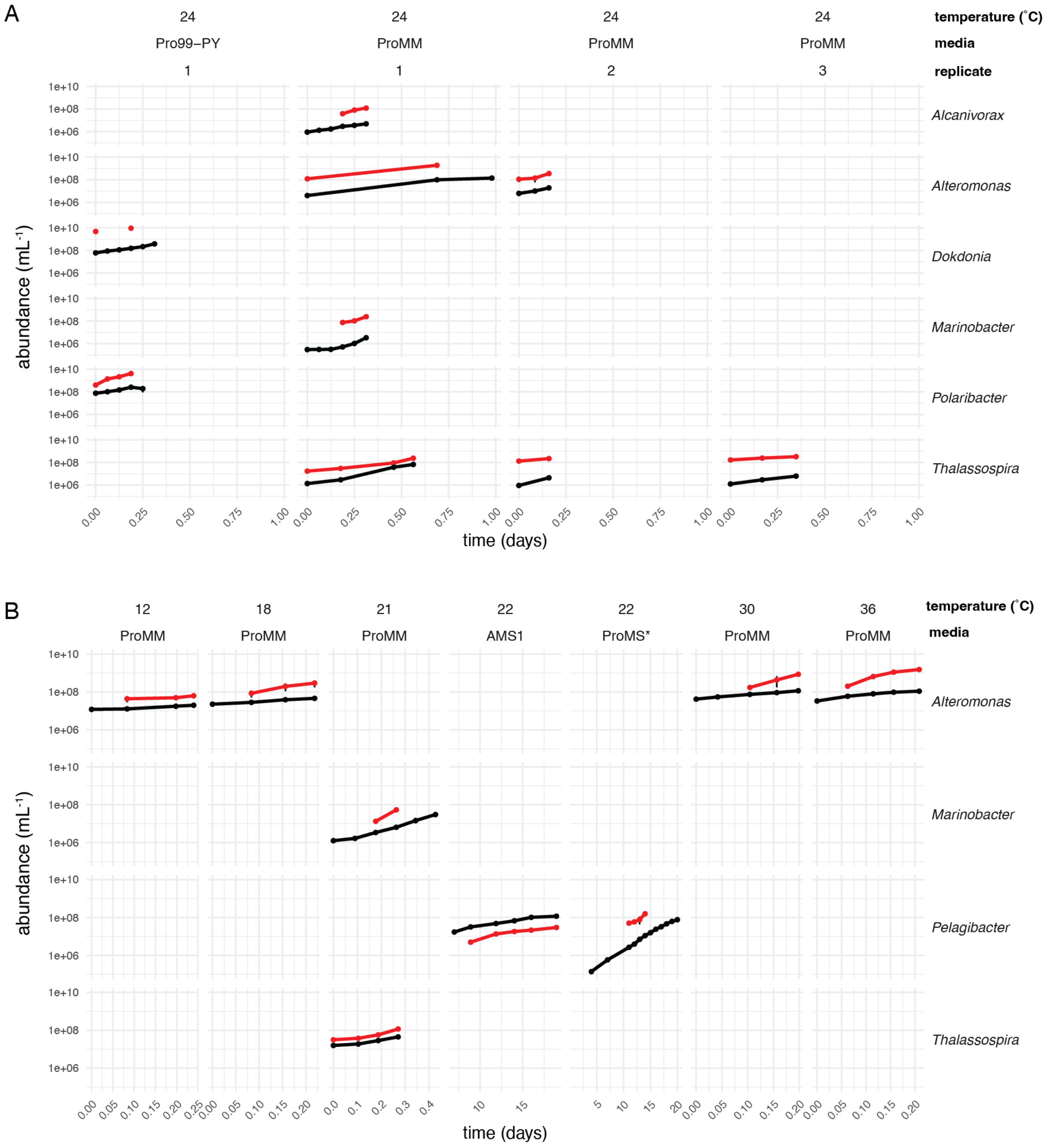


**FIG S1** Vesicle production in marine heterotrophs. Plots (A-B) represent abundance of cells (black) and vesicles (red) in the strain (noted at right) under the specified conditions. Values indicate the mean (± SD) from biological triplicates.

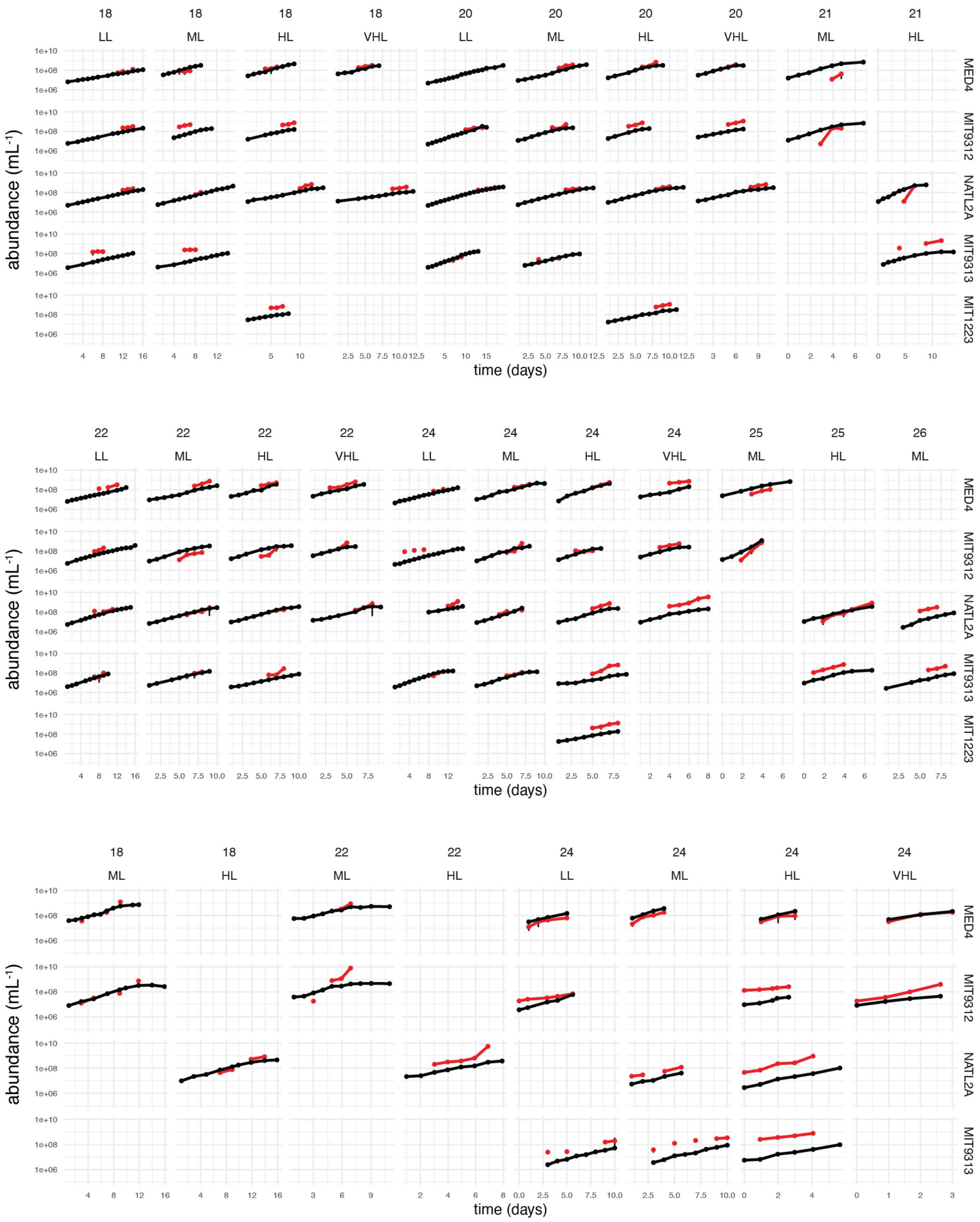


**FIG S2** Vesicle production in *Prochlorococcus*. Plots represent abundance of cells (black) and vesicles (red) in the strain (noted at right). Temperature (ºC) and relative light level is indicated at the top of each set of plots. Values indicate the mean (± SD) from biological triplicates. Light levels: LL= low light, ML= medium light, HL= high light, VHL=very high light (see Table S1).

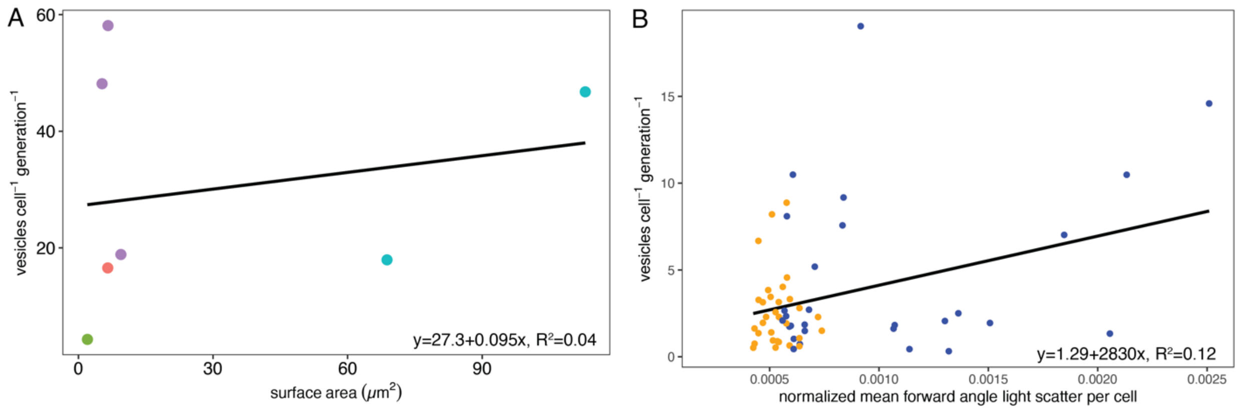


**FIG S3** Relationship between cell size and vesicle production rates. (A) Cell surface area versus median vesicle production rate across the different marine microbes shown in Fig. 1A (at 24 °C). Colors represent taxonomic groupings of microbes at the Class level: Cyanophyceae (green), Alphaproteobacteria (red), Gammaproteobacteria (purple), Flavobacteriia (blue). (B) Flow-cytometry measurements of mean forward angle light scatter per cell (a proxy for cell size), normalized to 2 µm internal standard beads, versus vesicle production rate among *Prochlorococcus* grown at different combinations of light and temperature. Colors indicate whether that cell belonged to a high light (HL)-adapted (orange) or low light (LL)-adapted (blue) *Prochlorococcus* ecotype.

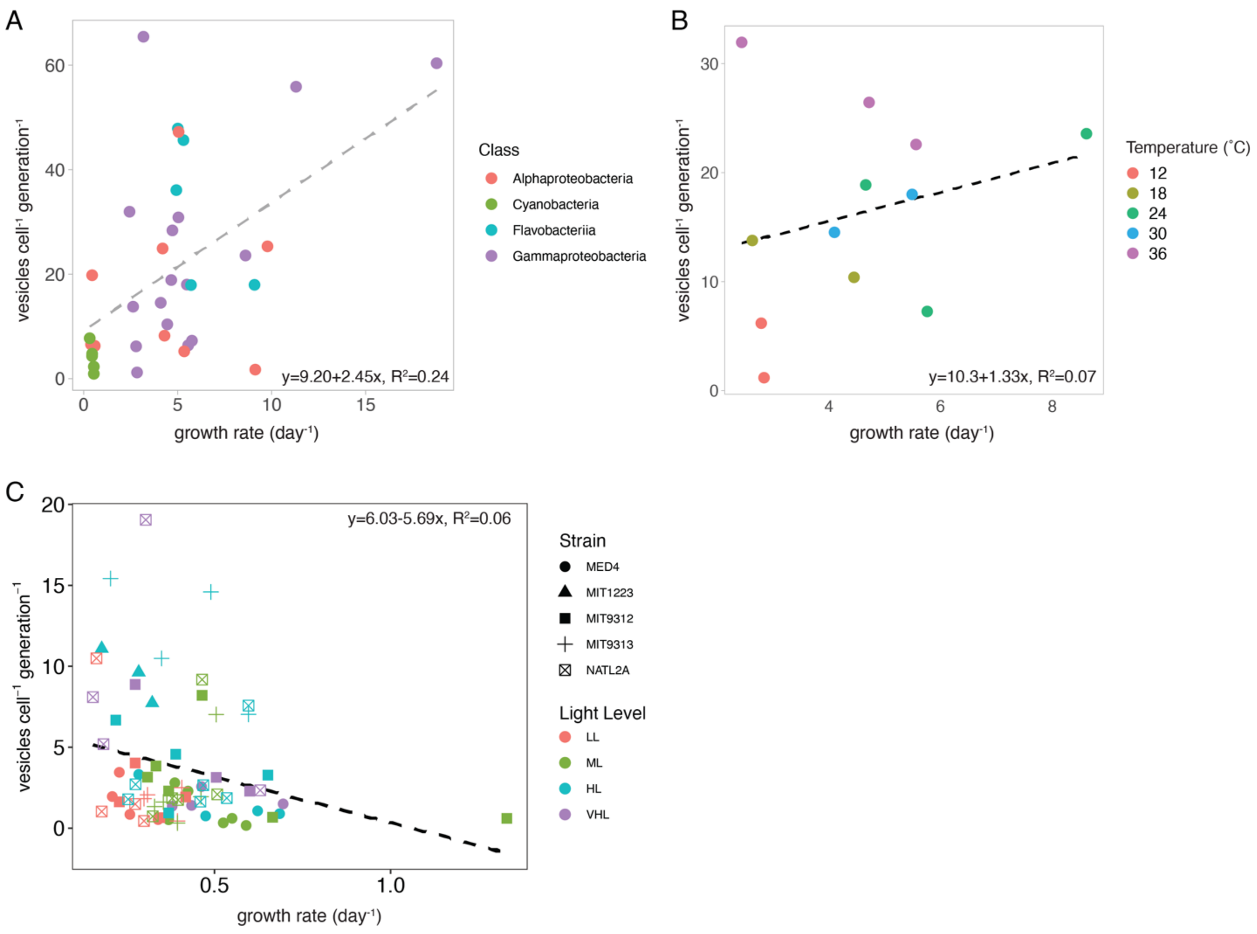


**FIG S4** Relationship between growth rate and vesicle production across (A) All marine taxa and conditions shown in Fig. 1; (B) *Alteromonas* grown at different temperatures in Fig. 2B; (C) *Prochlorococcus* strains at all combinations of light and temperature tested. Light levels: LL= low light, ML= medium light, HL= high light, VHL=very high light (see Table S1). Dashed lines indicate the linear regression through each data set with equation noted.

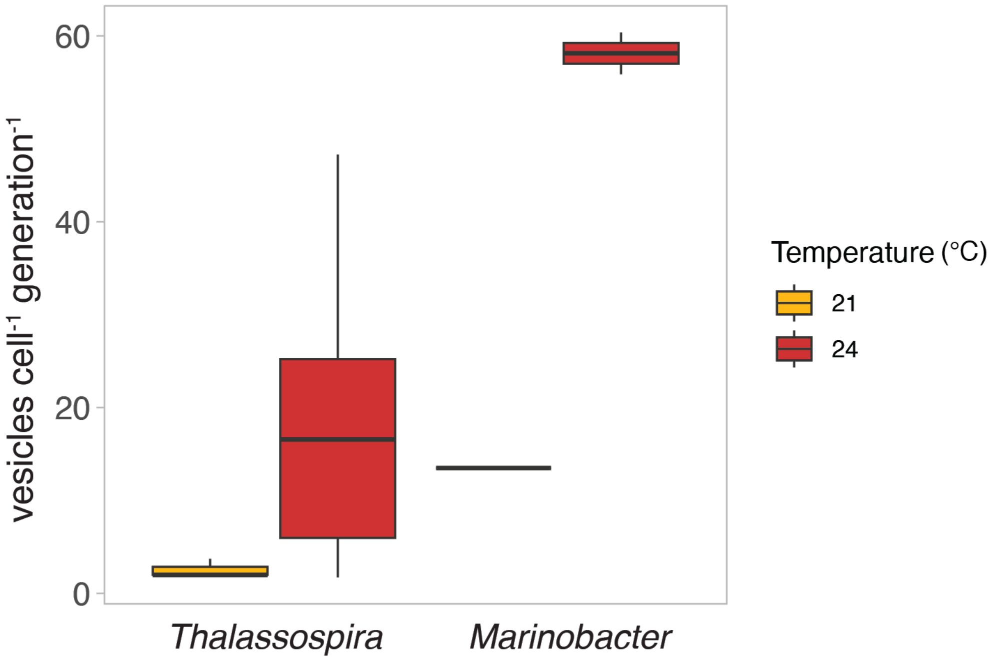


**FIG S5** Impact of temperature on vesicle production rates in *Thalassospira* and *Marinobacter*.

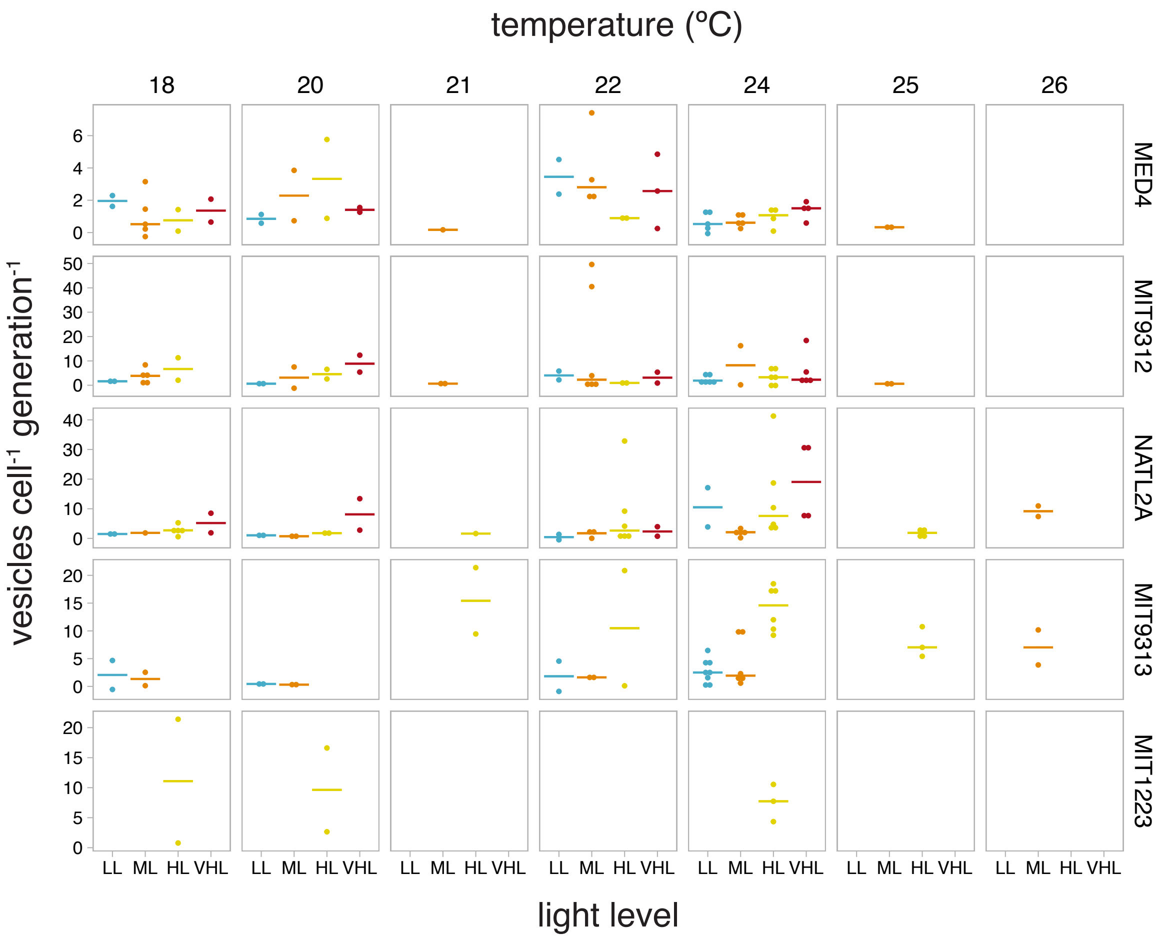


**FIG S6** Detailed *Prochlorococcus* vesicle production across different growth conditions. Production rate data across all combinations of strains (MED4, MIT9312, NATL2A, MIT9313, and MIT1223), light, and temperature. Horizontal lines indicate the median vesicle production rate measured for the indicated set of conditions. Light levels: LL= low light, ML= medium light, HL= high light, VHL=very high light (see Table S1).

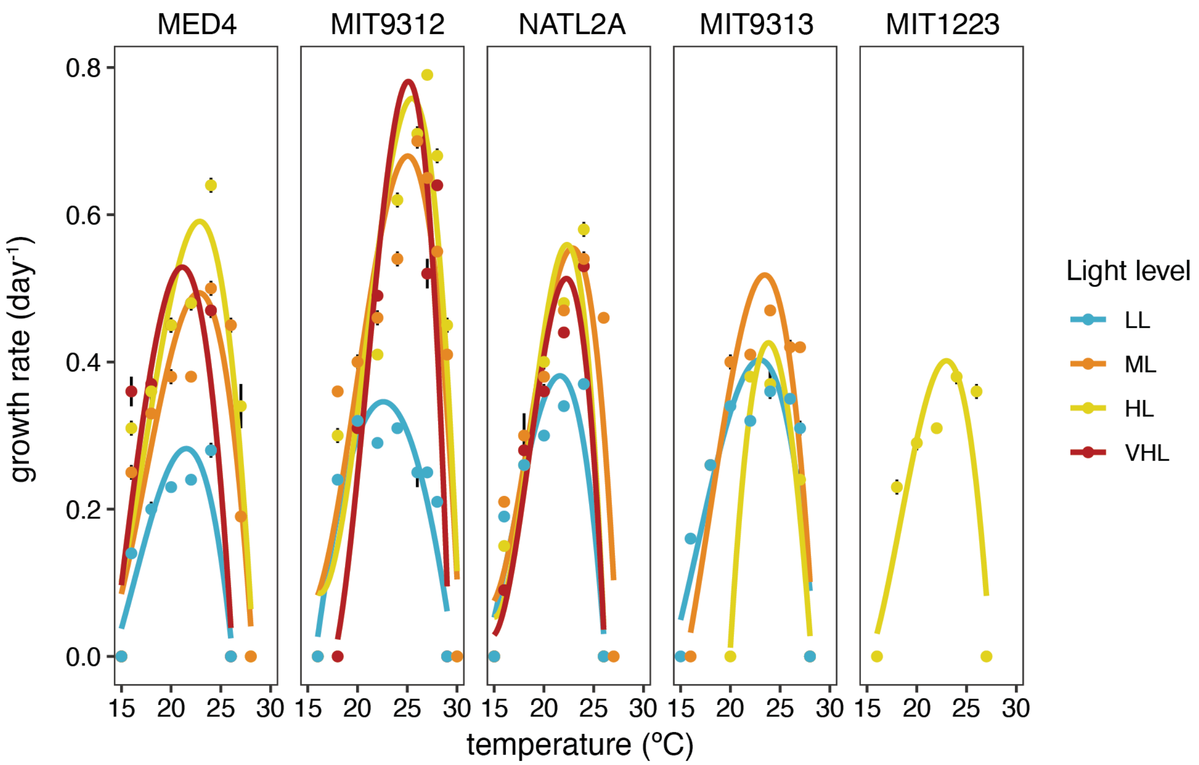


**FIG S7** Temperature growth optima of *Prochlorococcus* strains. Values indicate measured growth rates for axenic cultures grown across different strain-specific light levels at the indicated temperature. Light levels: LL= low light, ML= medium light, HL= high light, VHL=very high light. Details on light levels and numerical values are found in Table S1.

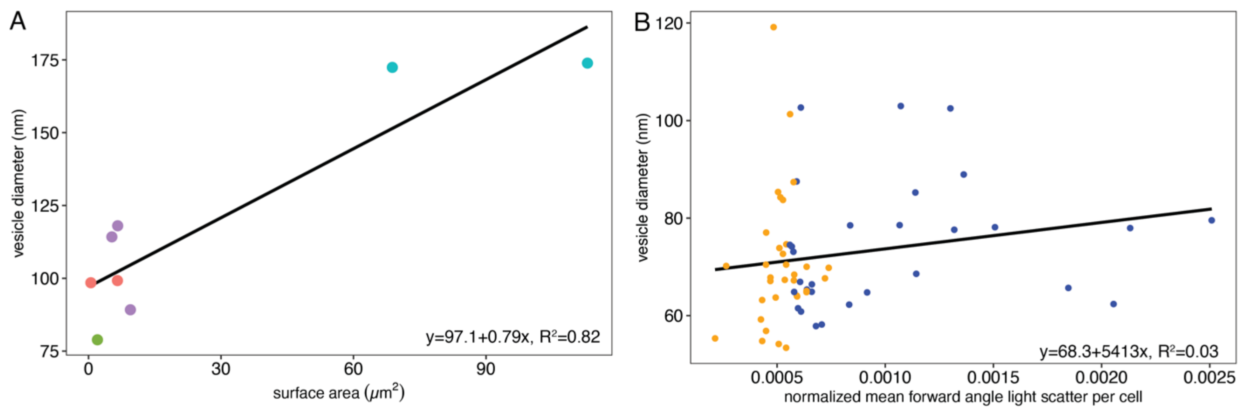


**FIG S8** Relationship between cell and vesicle sizes. (A) Cell surface area versus vesicle size across the different marine microbes shown in Fig. 1A (at 24 °C). Colors represent taxonomic groupings of microbes at the Class level: Cyanophyceae (green), Alphaproteobacteria (red), Gammaproteobacteria (purple), Flavobacteriia (blue). (B) Flow-cytometry based measurements of mean forward angle light scatter per cell (a proxy for cell size), normalized to 2 µm internal standard beads, versus vesicle diameter among *Prochlorococcus* grown at different combinations of light and temperature. Colors indicate whether that cell belonged to a high light (HL)-adapted (orange) or low light (LL)-adapted (blue) *Prochlorococcus* ecotype.

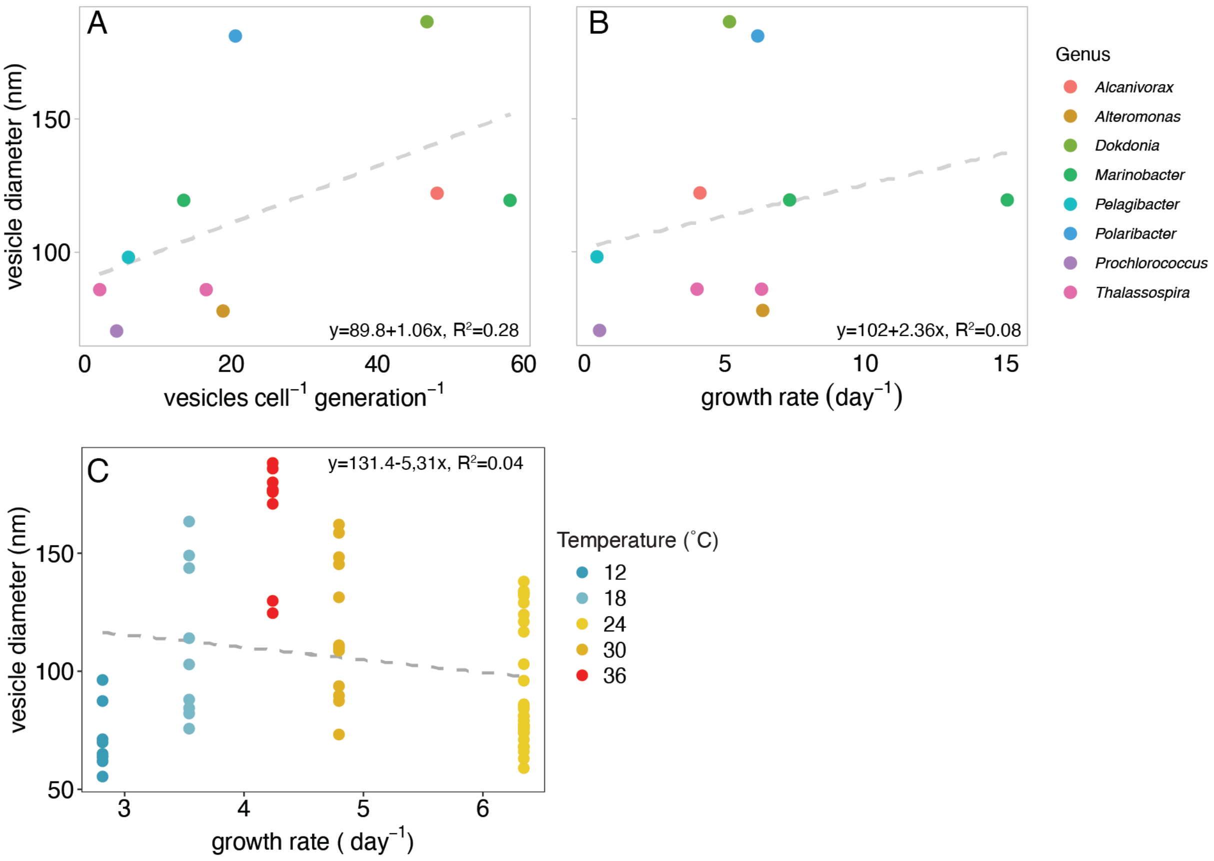


**FIG S9** Associations between vesicle size and culture parameters. Relationship between vesicle diameter and (A) vesicle production rate or (B) cellular growth rate across all taxa indicated at right. Regression analysis indicated a positive association between vesicle diameter and both parameters as indicated, but neither correlation was statistically significant (Pearson correlation, $p > 0.05$). (C) Relationship between vesicle diameter and cellular growth rate across *Alteromonas* strains grown at different temperatures.

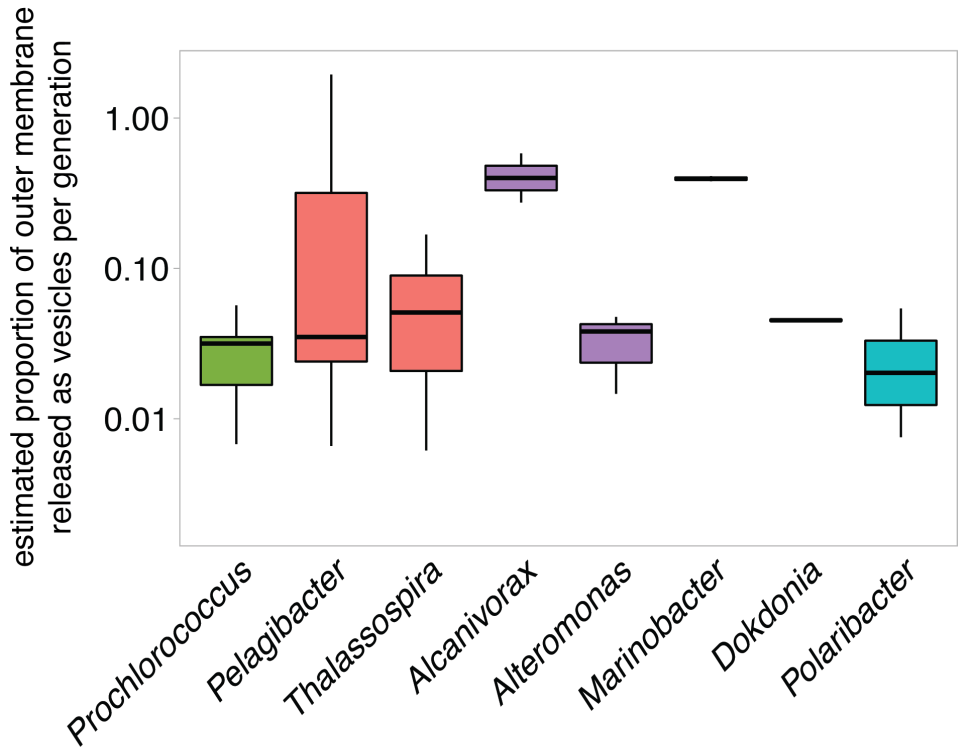


**FIG S10** Cellular resource investment in vesicles across taxa. Relative cellular cost of vesicle production is estimated based on total vesicle surface area released per generation as a proportion of cellular surface area. Vesicle production rate ranges are based on data from Figure 1A-B. Colors represent taxonomic groupings of microbes at the Class level: Cyanophyceae (green), Alphaproteobacteria (red), Gammaproteobacteria (purple), Flavobacteriia (blue).
